# Equivalent clinical outcome after vitrified‐thawed blastocyst transfer using semi‐automated embryo vitrification system compared with manual vitrification method

**DOI:** 10.1002/rmb2.12320

**Published:** 2020-02-26

**Authors:** Atsuko Miwa, Yukiko Noguchi, Kayo Hosoya, Yuusuke Mori, Takuma Sato, Yuta Kasahara, Miwa Hidaka, Hiroshi Hayashi

**Affiliations:** ^1^ Keiai Reproductive & Endosurgical Clinic Wako‐shi Japan; ^2^ Department of Obstetrics and Gynecology The Jikei University School of Medicine Minato‐ku Japan

**Keywords:** automated vitrification, blastocyst, cryopreservation, Gavi^®^, pregnancy

## Abstract

**Purpose:**

This study compared Gavi^®^, an automated system for the equilibration and dehydration steps of vitrification, and a manual vitrification procedure in terms of effects on clinical outcomes.

**Methods:**

The authors retrospectively compared survival rate, and clinical and perinatal outcomes after vitrified‐thawed single blastocyst transfer between Gavi^®^ (G method) in 398 cases and Cryotop^®^ (C method) in 208 cases.

**Results:**

With C and G methods, survival rates were 98.6% (208/211) and 99.3% (398/401), total pregnancy rates were 34.3% (72/208) and 33.4% (133/398), and total miscarriage rates were 22.2% (16/72) and 24.8% (33/133), respectively. Among women <35 years old, pregnancy rates were 41.1% (30/73) and 40.5% (62/153) and miscarriage rates were 13.3% (4/30) and 16.1% (10/62) with C and G methods, respectively. Among women ≥35 years old, pregnancy rates were 31.1% (42/135) and 29.0% (71/245) and miscarriage rates were 28.6% (12/42) and 32.4% (23/71) with C and G methods, respectively. C and G methods showed no significant differences in any trials, including gestational age, cesarean section rate, or birthweight (*P* > .05 each).

**Conclusions:**

Gavi^®^ showed comparable clinical outcomes to the manual vitrification method and can be considered an alternative vitrification procedure in assisted reproductive technology.

## 1 INTRODUCTION

The history of embryo freezing started with a report in 1972 by Whittingham et al,^1^ showing that mouse blastocysts survived a freeze‐thaw cycle. Since then, the need for embryo freezing in assisted reproductive technology (ART) has gained recognition. Improvements of embryo culture and cryopreservation methods have increased the performance of vitrified embryo transfer through better embryo selection, to the point of providing almost the same results as fresh embryo transfer.

Two basic methods are available for the cryopreservation of human oocytes and embryos: slow freezing and vitrification. Currently, vitrification has emerged as the more reliable method for cryopreservation. As a result, fewer blastocysts are transferred, allowing reductions in the frequency of multiple pregnancies and an increased chance of healthy transplant.

To preserve embryos in good condition, it is necessary to maintain (a) reversible metabolic arrest in the embryo; (b) the structure of the embryo itself (including DNA); (c) an acceptable survival rate; and (d) normal embryo growth after thawing. Furthermore, the cryopreservation technique must be stable and allow high reproducibility. A key problem in embryo cryopreservation is to avoid the formation of ice crystals in cells, as these can cause physical and chemical damage to the embryo.

Vitrification involves preventing the formation of ice crystals inside cells and freezing water in a glassy state to arrest molecular conversions without inducing any structural reorganization. Vitrification is defined as a method for solidification of a liquid by raising its consistency in the process of freezing at an extremely low temperature.^2^, ^3^ Generally, a vitrification solvent comprises a cryoprotectant that remains unfrozen even with quite rapid cooling to an ultra‐low temperature. In addition, freezing and thawing speeds show inverse correlations with cryoprotectant concentration in the vitrification process.

Seki et al^4^, ^5^ reported that thawing speed plays a more important role than freezing speed in the survival rate of mouse oocytes. In 1985, Rall et al^6^ first reported the blastocyst freezing method without ice crystals. In 2000, Yoon et al reported a healthy pregnancy and live birth of a human embryo using vitrification.^7^, ^8^ By preventing ice crystal formation, blastocysts have shown an extended survival rate after thawing compared to programmable rate freezing used in ART.^9^ In fact, clinics familiar with vitrification reportedly show around a 90% blastocyst recovery rate and pregnancy and live birth rates equal to or higher than those with fresh embryo transfer.^10^, ^11^, ^12^

The vitrification method is very simple and does not require particularly expensive equipment, and the blastocysts are placed in a very small amount of vitrification solution that can be frozen quickly, compared with traditional closed straw or vial devices. However, the toxicity of high concentration of cryoprotectant can present an obstacle to embryologists unfamiliar with vitrification technology. Basically, the vitrification media is more toxic than any cryopreservation media used in slow freezing methods. This places practical limitations on the speed of freezing used to attain vitrification and also biological limitations on the concentration of cryoprotectant in cells.

While vitrification is a widely accepted cryopreservation method in ART, the actual procedure requires the manual performance of several steps by the embryologist and requires a long learning curve to obtain the necessary level of skill. Results are thus extremely dependent on the embryologist and clinic. For those reasons, ART clinics try to maintain trained and skillful embryologists for consistent results.^13^, ^14^, ^15^

Recently, many vitrification devices and protocols have become available, varying methods such as using different types and concentrations of cryoprotectant, different temperatures and timings of exposure of the embryo, different freezing and thawing speeds, and even whether the embryo comes into direct contact with liquid nitrogen. At present in Japan, a manual non‐closed‐type embryo cryopreservation method using Cryotop^®^ (C method; Kitazato Corporation) is mainly used as the vitrification method. C method offers a high freezing speed and high embryo survival rate. However, because the embryo comes into direct contact with liquid nitrogen, there are risks of infection and contamination, and advanced techniques are required for vitrification and thawing procedures. The possibility of variations in performance and human error has also been pointed out. In addition, the work burden of the vitrification and thawing steps is considered problematic for embryologists, and automation of the vitrification process has thus been expected to obtain standardization of high reproducibility for optimal performance.

Gavi^®^ (G method; Merck Biopharma) is a semi‐automated closed‐type vitrification system in equilibration and is available for vitrification of oocytes, cleavage‐stage embryos, and blastocysts. G method was first developed by Genea and Planet Innovation. The Gavi system controls multiple vitrification steps and concentrations of vitrification medium that are difficult to control manually. Gavi^®^ can also control the timing, temperature, and duration of exposure of the vitrification medium to oocytes, embryos, and blastocysts.

By automating embryo vitrification with Gavi^®^, it is considered to eliminate variations in results due to the skill of the embryologists and human error, and to shorten the working hours of the embryologists, so that even a facility with small number of embryologists can expect improving work flow in vitrification procedure and also consistent clinical outcome.

The automated Gavi system has thus been expected to offer comparable performance to the current gold standard C method.^16^, ^17^

G method is able to vitrify four blastocysts at once, and the procedure is standardized, so little variation between practitioners is considered to be present in the equilibration step.

Our clinic introduced a semi‐automated vitrification method using G method in May 2017. Performance was expected to be stably maintained, but concerns were raised that the embryo survival rate might be adversely impacted by the slow freezing rate in the closed‐type system (Table 1). This study therefore examined the effects of this semi‐automated vitrification system on survival rate after thawing, pregnancy rate, miscarriage rate, and perinatal outcomes compared with the manual vitrification method.

**Table 1:** Comparison of characteristics between Cryotop and Gavi

	C method	G method
Variation in facility/embryologists	Large	Very small
Learning time	Long	Short
Human error possibility	Moderate	Low
Contamination risk	Possible	Low
Vitrification speed	28 000°C/min	16 000°C/min
Vitrification cost	Low	Expensive

## 2 MATERIALS AND METHOD

### 2.1 Study design

After obtaining sufficient informed consent, blastocysts were vitrified by C method in 181 patients of 208 cases from February to April 2017 and by G method in 302 patients of 398 cases from June to December 2017. Right after thawing, blastocyst survival rate was compared between C method and G method.

In C and G methods, patients were diagnosed as ART indication because of oviductal factor, male factor, ovulation factor, implantation failure, and unexplained infertility.

After single vitrified‐thawed blastocyst transfer, we retrospectively compared pregnancy rate, miscarriage rate, gestational age, birthweight, and cesarean section rate.

### 2.2 Ovarian stimulation

Patients were stimulated by controlled ovarian stimulation protocol (GnRH antagonist, short GnRH agonist, or long GnRH agonist) or modified mild ovarian stimulation protocol (clomiphene citrate, letrozole). For final maturation stimulation, patients were administered human chorionic gonadotropin (hCG) or GnRH agonist when dominant follicles reached a diameter of 18 mm.

### 2.3 In vitro fertilization and embryo culture

Follicular aspiration was performed by vaginal ultrasonography at 36 hours after hCG or GnRH agonist administration. The oocytes were inseminated using either conventional IVF or intracytoplasmic sperm injection depending on the semen parameters and incubated in Continuous Single Culture^™^ (FUJI FILM Irvine Scientific) medium until Day 5.

In our clinic, the blastocyst vitrification is conducted following Gardner's criteria with early stage blastocysts classified 1 or 2, or that with more than CC grade in Day 5 and good morphology blastocysts are considered more than equal 3BB grade.

Clinical pregnancy was defined as recognition of a gestational sac on transvaginal ultrasonography at around gestational week 5. Miscarriage was defined as spontaneous abortion by gestational week 10.

### 2.4 Blastocyst vitrification and thawing

**Figure 1:**
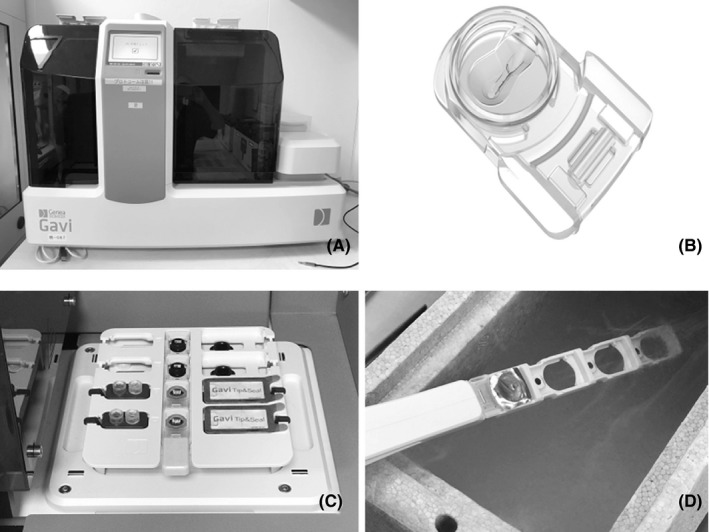
Images of the Gavi system and its consumable components. A, Appearance of the whole Gavi system with the automated pipette inside. B, Gavi pod, which can preserve oocyte, embryo, and blastocyst on a slit in the pod. C, Vitrification media, cassette, and disposable tip and seal. D, After the dehydration step, the Gavi pod is dumped into liquid nitrogen to complete the vitrification step

Gavi^®^ can automate the procedure for equilibration and dehydration steps in vitrification (Figure 1A). In Gavi, the equilibration and dehydration process is conducted in a closed‐type vitrification device called a “Gavi pod” (Figure 1B). After dehydration, Gavi pod is put on “cassette” as an attachment holding up Gavi pods. This cassette containing Gavi pod is then dumped into liquid nitrogen storage for completion of vitrification (Figure 1C,D).

The vitrification step using Gavi^®^ was conducted following the protocol specified by each manufacturer. Briefly, blastocysts were held in a slit on the Gavi pod with a small amount of HEPES buffered medium, and the pod was then attached to the cassette. The embryologist then pressed the button to start blastocyst vitrification mode. All steps of discarding or adding solvent were automatically conducted with disposable sterilized tips. HEPES buffer medium was discarded, and the equilibration solvent was continuously added. After equilibration steps, the solvent was discarded and the dehydration step was conducted. After discarding the dehydration medium, each Gavi pod was heat‐sealed to a lid and the cassette was stored in a liquid nitrogen storage unit before being placed in a liquid nitrogen tank. For embryologists, this protocol is very simple: set the embryo in the Gavi pod, press the start button, and after the dehydration step, dump the cassette containing the Gavi pods into liquid nitrogen storage.

For C and G methods, vitrified blastocysts were thawed using Cryotop thawing kit VT506 (Kitazato Corporation) and Gems Warming Set (Genea), respectively, following the manufacturer's protocol. Briefly, for C method, the Cryotop^®^ was removed from the protective cover in liquid nitrogen, and the end of the strip was placed directly into 1.0 mL of 37°C thawing solution for 1 minute. The blastocysts were subsequently transferred into 500 μL of diluent solution for 3 minutes and washed twice with 500 μL of washing solution for 5 minutes at room temperature.

For G method, Gems Warming Solution 1 to 3 (Genea) was equilibrated to room temperature and set into four well dish (solution 3 was set in two wells). Gavi pod was retrieved from cassette under liquid nitrogen and immersed into 37°C of deionized water in water bath for 2 seconds and removed water by a wipe. Under microscope, the lid seal of Gavi pod was removed and added 10 μL of solution 1 for 1 minutes. Thereafter, the blastocysts were transferred into solution 2 for 3 minutes and subsequently transferred twice into solution 3 for 5 and 1 minutes, respectively.

After thawing, all blastocysts were cultured in blastocyst medium for up to 4 hours and if greater than 50% of the cells were intact and re‐expansion had been observed, blastocysts were considered as survived. All vitrified‐thawed operations with C method and G method in this study were conducted by the same embryologist, who had experience conducting more than 1000 cases using the C method before the start of this study.

### 2.5 Embryo transfer

For embryo transfer, endometrium preparation was conducted by the hormone‐replacement therapy. Briefly, transdermal E2 (Estrana^®^; Hisamitsu Pharmaceutical) was administered from days 1‐3 of menstruation cycle, and after the endometrial thickness was reached at least 6.5 mm, progesterone (UTOROGESTAN vaginal capsules^®^; Fuji Pharmaceutical) was administered until 9 weeks of pregnancy.

After thawing, single blastocyst was transferred into uterus after 6 days of progesterone treatment using Kitazato ET catheter^®^ (Kitazato Corporation) under ultrasound guidance.

All the blastocysts performed laser‐assisted hatching before embryo transfer. The presence of a gestational sac was confirmed via transvaginal ultrasonography at around 5 weeks. Pregnancy and miscarriage rates were analyzed for the overall cohort, and also for the <35‐year‐old and ≥35‐year‐old patient subgroups.

### 2.6 Statistical analysis

Statistical analysis was conducted using chi‐square test for patient's proportion, survival rate, pregnancy rate, miscarriage rate, and cesarean section rate. For other parameters, Student's *t* test was applied for statistical analysis. *P*‐value <.05 was considered significant.

## 3 RESULTS

### 3.1 Background characteristics of patients

With C method, total number of patients was 181, 66 < 35 years old and 115 ≥ 35 years old, and mean patient age was 36.1 years old, with 73 cases <35 years old and 135 cases ≥35 years old. The total number of embryos transferred was 208, and the total number of blastocysts vitrified and thawed was 211.

With G method, total number of patients was 302, 118 < 35 years old and 184 ≥ 35 years old, and mean patient age was 36.0 years old, with 153 cases <35 years old and 245 cases ≥35 years old. The total number of embryos transferred was 398 and the total number of blastocysts vitrified and thawed was 401. No significant differences in any of these characteristics were apparent between groups (Table 2).

**Table 2:** Comparison of patient background characteristics between Cryotop and Gavi

	C method	G method
No. of blastocysts thawed	211	401
No. of embryo transfers	208	398
Mean patient age (±SD)*	36.12 ± 4.6	36.06 ± 4.7
No. <35 y (%)*	73 (35.1%)	153 (38.4%)
No. ≥35 y (%)*	135 (64.9%)	245 (61.6%)

* No significant difference in patient age was observed between Cryotop and Gavi (*P* > .05).

The percentage of good morphology blastocyst rate in G and C methods was 77.5% (311/401) and 81.3% (170/209), in <35‐year‐old patients was 85.6% (131/153) and 90.4% (66/73), and in ≥35‐year‐old patients was 72.6% (180/248) and 76.5% (104/136), respectively. There was no significant difference in the percentages of good morphology blastocyst between the two groups.

The proportion of Gardner's criteria 1 or 2 for G method was 15.7% (63/401) in whole age, 9.8% (15/153) in <35 years old, and 19.6% (48/248) in ≧35 years old and for C method was 14.4% (30/209) in whole age, 8.2% (6/73) in <35 years old, and 17.6% (24/136) in ≧35 years old, respectively.

### 3.2 Blastocyst survival rate

The blastocyst survival rate after thawing was 98.6% (208/211) with C method and 99.3% (398/401) with G method, showing no significant difference (Table 3).

**Table 3:** Comparison of survival rate after thawing blastocysts between Cryotop and Gavi

	C method	G method
No. survived/no. thawed	208/211	398/401
Survival rate	98.6%	99.3%

Note

No significant difference was observed between Cryotop and Gavi in survival rate (*P* > .05).

### 3.3 Pregnancy and miscarriage rates

**Figure 2:**
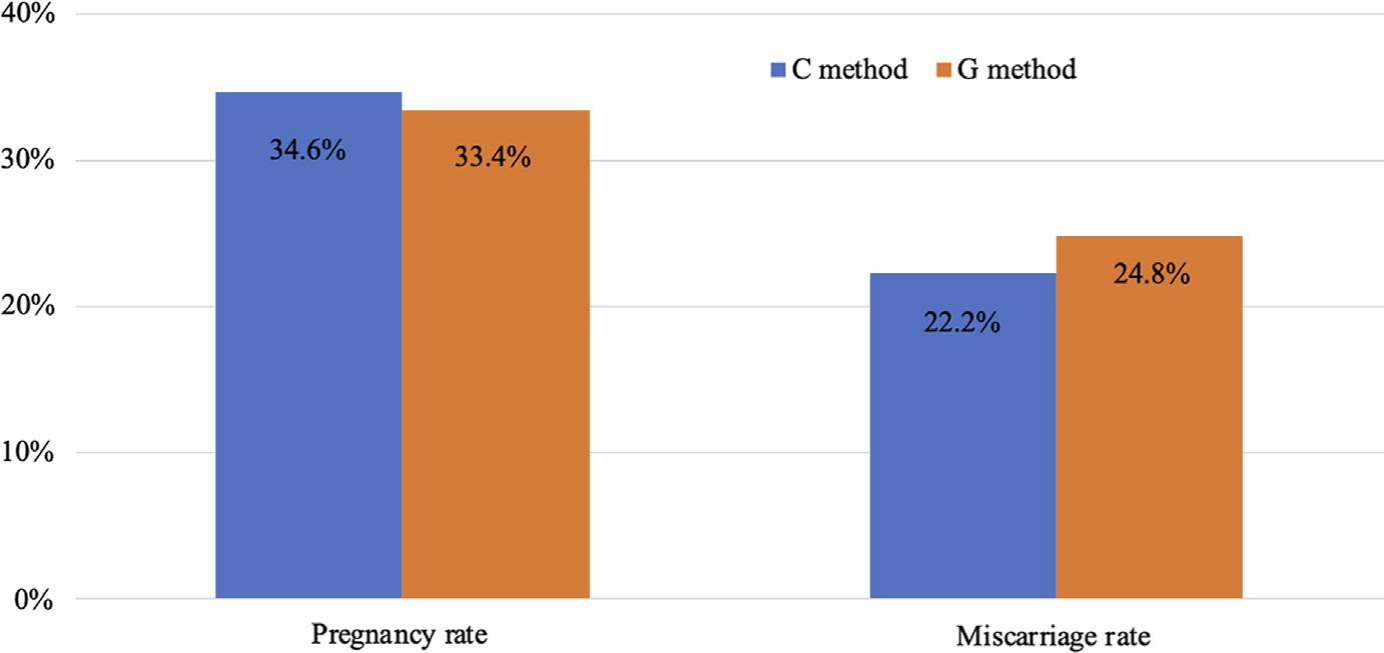
Comparison of pregnancy and miscarriage rates between Cryotop and Gavi for total cohort

For the total cohort, the pregnancy rates were 34.6% (72/208) for C method and 33.4% (133/398) for G method, and miscarriage rates were 22.2% (16/72) for C method and 24.8% (33/133) for G method (Figure 2).

**Figure 3:**
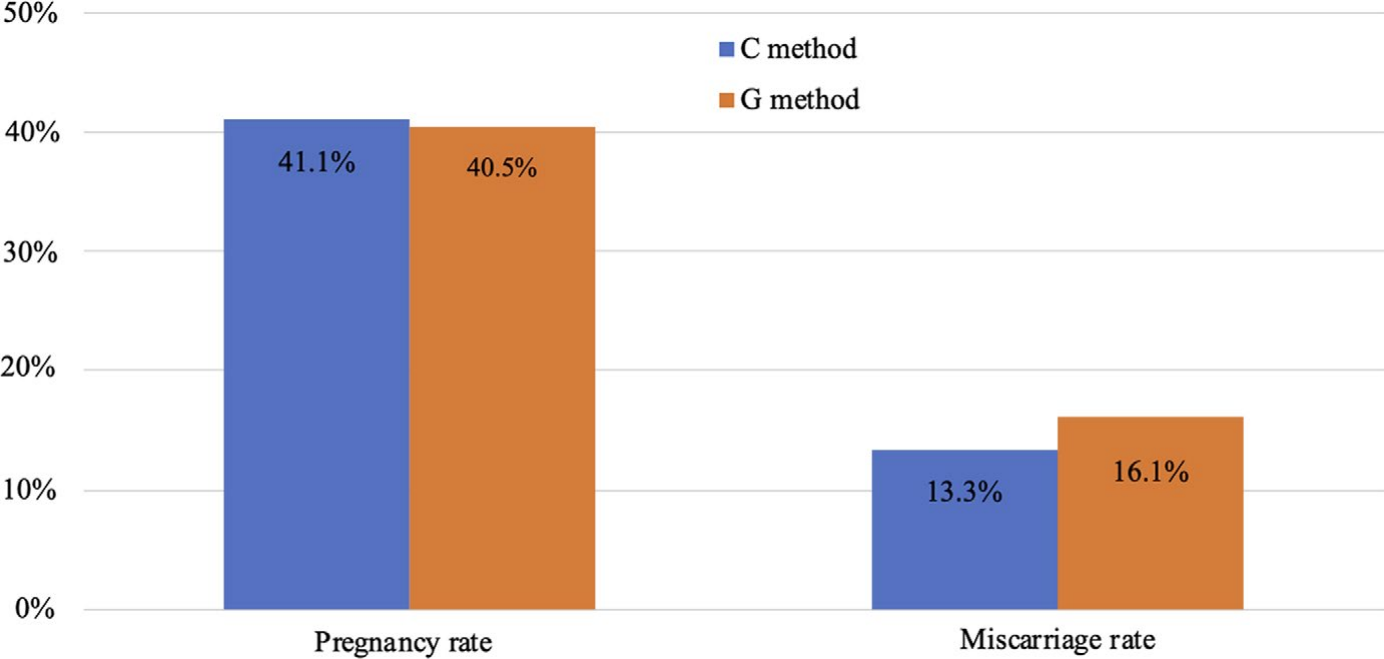
Comparison of pregnancy and miscarriage rates between Cryotop and Gavi in patients <35 y old

In patients <35 years old, pregnancy rates were 41.1% (30/73) for C method and 40.5% (62/153) for G method, and miscarriage rates were 13.3% (4/30) for C method and 16.1% (10/62) for G method (Figure 3).

**Figure 4:**
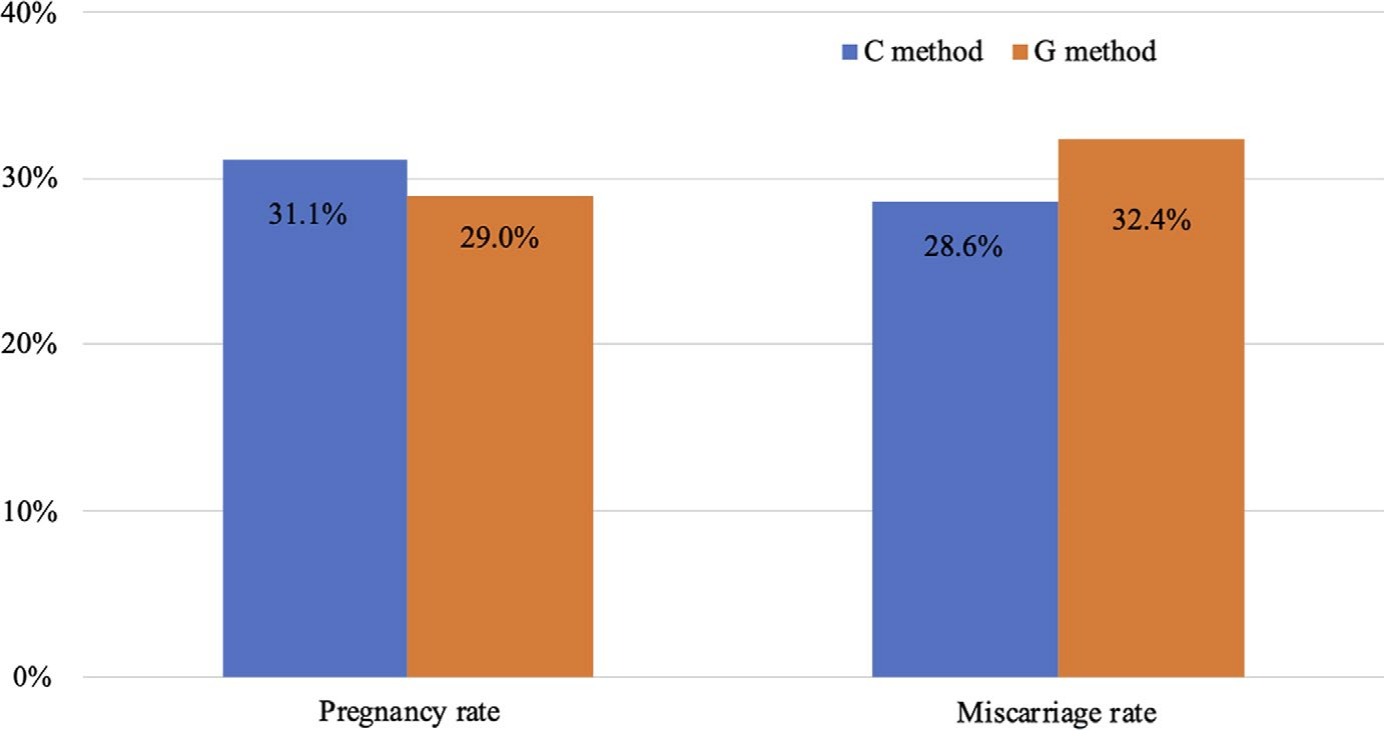
Comparison of pregnancy and miscarriage rates between Cryotop and Gavi in patients ≥35 y old

In patients ≥35 years old, pregnancy rates were 31.1% (42/135) with C method and 29.0% (71/245) with G method, and miscarriage rates were 28.6% (12/42) with C method and 32.4% (23/71) with G method (Figure 4).

No significant differences in pregnancy or miscarriage rates were evident between C and G methods for any age‐groups.

For Gardner's criteria 1 or 2, pregnancy rate for G method was 17.5% (11/63) in whole age, 20% (3/15) in <35 years old, and 16.7% (8/48) in ≧35 years old and for C method was 16.7% (5/30) in whole age, 33.3% (2/6) in <35 years old, and 12.5% (3/24) in ≧35 years old, and, on the other hand, miscarriage rate for G method was 27.3% (3/11) in whole age, 0% (1/3) in <35 years old, and 37.5% (3/8) in ≧35 years old and for C method was 20.0% (1/5) in whole age, 0.0% (0/2) in <35 years old, and 33.3% (1/3) in ≧35 years old, respectively. There was no significant difference between C and G methods in each age‐group.

For Gardner's criteria ≧3, pregnancy rate for G method was 36.1% (122/338) in whole age, 42.8% (59/138) in <35 years old, and 31.5% (63/200) in ≧35 years old and for C method was 37.4% (67/179) in whole age, 41.8% (28/67) in <35 years old, and 34.8% (39/122) in ≧35 years old, respectively, and, on the other hand, miscarriage rate for G method was 24.6% (30/122) in whole age, 16.9% (10/59) in <35 years old, and 31.7% (20/63) in ≧35 years old and for C method was 22.4% (15/67) in whole age, 14.3% (4/28) in <35 years old, and 28.2% (11/39) in ≧35 years old, respectively. There was no significant difference between C and G methods in each age‐group.

### 3.4 Perinatal outcomes

We also examined perinatal outcomes following vitrified‐thawed single blastocyst transfer (Table 4). With C method, total follow‐up data for perinatal outcomes were collected from 30 cases, with a mean gestational age of 38 weeks 6 days, a cesarean section rate of 46.7%, and a mean birthweight of 3103 g. With G method, total follow‐up data for perinatal outcomes were collected from 36 cases, with a mean gestational age of 39 weeks 4 days, a cesarean section rate of 41.6%, and a mean birthweight of 3226 g. In this study, no multiple pregnancy was observed in G and C methods.

**Table 4:** Comparison of perinatal outcomes between Cryotop and Gavi

	C method	G method	*P*‐value
Cases	30	36	
Gestational age	38 wk 6 d	39 wk 4 d	N.S
Birthweight (g)	3103 ± 512	3226 ± 352	N.S
Cesarean section rate (%)	46.7	41.4	N.S

Note

No significant difference was observed between Cryotop and Gavi in gestational age, birthweight, or cesarean section rate (*P* > .05 each).

No significant differences in gestational age, cesarean section rate, or birthweight were evident between C and G methods.

## 4 DISCUSSION

In this study, survival rate, pregnancy rate, and miscarriage rate after vitrified‐thawed single blastocyst transfer were compared between C and G methods. No significant differences were observed between the two groups. Similarly, no significant differences in perinatal outcomes were observed between groups.

Closed‐type devices tend to show slower freezing speeds, compared with open‐type devices, but Roy et al showed that the recovery rate for blastocysts in mice was not significantly impacted with Gavi^®^. Gavi^®^ showed no significant differences compared with C method; however, sample numbers were small in that study. In addition, recovery and survival rates of zygotes, cleavage‐stage embryos, and complete hatched blastocysts were also studied in that study, and recovery rates did not differ significantly for each stage. In terms of survival rate, even though zygotes and blastocysts showed almost equal results, cleavage‐stage embryo showed slightly lower results and complete hatched blastocysts showed significantly lower results for C method, compared with G method.^17^

Many reports have found no significant difference in clinical performance between non‐closed‐ and conventional closed‐type freezing methods, even though increasing the freezing speed from −2000 to −20 000°C/min has been reported to reduce the possibility of ice crystal formation.^18^ Freezing speed in closed‐type systems is considered to be slower than in open‐type systems. For example, the freezing speeds of closed‐type vitrification devices have been reported as −1220°C/min for Rapid‐i^®^, −1300°C/min for Vitrisafe^®^, and −4460°C/min for in‐straw dilution, whereas freezing speeds for open‐type devices have been reported as −15 000°C/min for Cryoloop^®^, −16 340°C/min for open‐pulled straw, and −22 800°C/min for Cryotop^®^.^19^, ^20^, ^21^

With G method, freezing speed is about −14 100°C/min, which is considerably improved compared to the conventional closed‐type freezing method. The effect of freezing speed on blastocyst viability is therefore considered small and as a result may not have affected clinical outcomes in this study.

We also examined perinatal outcomes. Dal et al^22^ described a case in which G method provided favorable pregnancy outcomes, and the present study showed similar results.

In addition, G method allows vitrification of four blastocysts and eight embryos or oocytes at once, and the required time was 15 minutes for blastocysts and 17 minutes for embryos and oocytes, respectively. The time required for multiple embryo vitrification thus considers to shorten, compared with one‐by‐one vitrification by C method, and Gavi^®^ may be able to reduce the workload of embryologists in vitrification.

However, learning times for each vitrification procedure were not examined in this study. Further study is thus necessary to clarify aspects of standardization and learning curve analysis in the vitrification procedure, comparing G and C methods.

Compared with conventional open‐type vitrification using Cryotop^®^, Gavi^®^ as a semi‐automated closed‐type embryo vitrification system did not significantly affect clinical outcome. The Gavi^®^ method can be considered as a useful method that may reduce variability in embryologist skill or facility protocols and equipment and may reduce the burden of the vitrification procedure in the ART laboratory.

In this study, we showed clinical and perinatal outcome in C and G methods; however, we did not study the effect of infertility cause or patient background on the clinical outcome of the different vitrification device. Further study is still necessary to clarify its efficacy and safety.

## DISCLOSURES

*Conflict of interest*: None of the authors have any commercial or financial involvement in connection with this work that represent or appear to represent any conflicts of interest. *Human rights statements and informed consent*: All procedures followed were in accordance with the ethical standards of the responsible committee on human experimentation (institutional and national) and with the Helsinki Declaration of 1964 and its later amendments. Informed consent was obtained from all patients for being included in the study. *Approval by ethics committee*: This study was approved by the medical ethics committees of Keiai Reproductive & Endosurgical Clinic (registration number: IRB2017‐001).

## References

[1] Whittingham DG , Leibo SP , Mazur P . Survival of mouse embryos, frozen to ‐196 ℃ and ‐289 ℃. Science. 1972;178:411‐414. 5077328

[2] Fahy GM , MacFarlane DR , Angell CA , Meryman HT . Vitrification as an approach to cryo‐preservation. Cryobiology. 1984;21:407‐426. 6467964 10.1016/0011-2240(84)90079-8

[3] Fahy GM . Vitrification: a new approach to organ cryopreservation In: Merryman HT, ed. Transplantation: Approaches to Graft Rejection. New York, NY: Alan R Liss; 1986:305‐335. 3540994

[4] Seki S , Mazur P . The dominance of warming rate over cooling rate in the survival of mouse oocytes subjected to a vitrification procedure. Cryobiology. 2009;59:75‐82. 19427303 10.1016/j.cryobiol.2009.04.012 PMC2729265

[5] Mazur P , Seki S . Survival of mouse oocytes after being cooled in a vitrification solution to ‐196 ℃ at 95° to 70,000 ℃/min and warmed at 610° to 118,000 ℃/min: a new paradigm for cryopreservation by vitrification. Cryobiology. 2011;62:1‐7. 21055397 10.1016/j.cryobiol.2010.10.159 PMC3041861

[6] Rall WF , Fahy GM . Ice‐free cryopreservation of mouse embryos at –196 ℃ by vitrification. Nature. 1985;313:573‐575. 3969158 10.1038/313573a0

[7] Kuleshova L , Gianaroli L , Magli C , Ferraretti A , Trounson A . Birth following vitrification of a small number of human oocytes: case report. Hum Reprod. 1999;14:3077‐3079. 10601099 10.1093/humrep/14.12.3077

[8] Yoon TK , Chung HM , Lim JM , Han SY , Ko JJ , Cha KY . Pregnancy and delivery of healthy infants developed from vitrified oocytes in a stimulated in vitro fertilization‐embryo transfer program. Fertil Steril. 2000;74:180‐181. 10899519 10.1016/s0015-0282(00)00572-0

[9] Walker DL , Tummon IS , Hammitt DG , Session DR , Dumesic DA , Thornhill AR . Vitrification versus programmable rate freezing of late stage murine embryos: a randomized comparison prior to application in clinical IVF. Reprod Biomed Online. 2004;8:558‐568. 15151720 10.1016/s1472-6483(10)61103-0

[10] Roy TK , Bradley CK , Bowman MC , McArthur SJ . Single‐embryo transfer of vitrified‐warmed blastocysts yields equivalent live‐birth rates and improved neonatal outcomes compared with fresh transfers. Fertil Steril. 2014;101:1294‐1301. 24582521 10.1016/j.fertnstert.2014.01.046

[11] Kato O , Kawasaki N , Bodri D , et al. Neonatal outcome and birth defects in 6623 single‐ tons born following minimal ovarian stimulation and vitrified versus fresh single embryo transfer. Eur J Obstet Gynecol Reprod Biol. 2012;161:46‐50. 22200255 10.1016/j.ejogrb.2011.12.005

[12] Takahashi K , Mukaida T , Goto T , Oka C . Perinatal outcome of blastocyst transfer with vitrification using cryoloop: a 4‐year follow‐up study. Fertil Steril. 2005;84:88‐92. 16009162 10.1016/j.fertnstert.2004.12.051

[13] Gosden R . Cryopreservation: a cold look at technology for fertility preservation. Fertil Steril. 2011;96:264‐268. 21718989 10.1016/j.fertnstert.2011.06.029

[14] Alpha Scientists in Reproductive Medicine . The Alpha consensus meeting on cryo‐ preservation key performance indicators and benchmarks: proceedings of an expert meeting. Reprod Biomed Online. 2012;25:146‐167. 22727888 10.1016/j.rbmo.2012.05.006

[15] Kader AA , Choi A , Orief Y , Agarwal A . Factors affecting the outcome of human blastocyst vitrification. Reprod Biol Endocrinol. 2009;7:99. 19758458 10.1186/1477-7827-7-99 PMC2757025

[16] Roy TK , Brandi S , Bradley CK , Peura TT . Automatic vitrification: development of the Gavi System In: Tucker MJ, Liebermann J, eds. Vitrification in Assisted Reproduction, 2nd edn. Boca Raton, FL: CRC Press; 2016:P61‐P68.

[17] Roy TK , Brandi S , Tappe NM , et al. Embryo vitrification using a novel semiautomated closed system yields in vitro outcomes equivalent to manual Cryotop method. Hum Reprod. 2014;29(11):2431‐2438. 25164022 10.1093/humrep/deu214

[18] Lane M , Schoolcraft WB , Gardner DK . Vitrification of mouse and human blastocysts using a novel cryoloop container‐less technique. Fertil and Steril. 1999;72:1073‐1078. 10593384 10.1016/s0015-0282(99)00418-5

[19] Desai NN , Goldberg JM , Austin C , Falcone T . The new Rapid‐i carrier is an effective system for human embryo vitrification at both the blastocyst and cleavage stage. Reprod Biol Endocrinol. 2013;11:41. 23672340 10.1186/1477-7827-11-41 PMC3660183

[20] Kuwayama M , Vajta G , Kato O , Leibo SP . Highly efficient vitrification method for cryopreservation of human oocytes. Reprod Biomed Online. 2005;11(3):300‐308. 16176668 10.1016/s1472-6483(10)60837-1

[21] Vanderzwalmen P , Ectors F , Grobet L , et al. Aseptic vitrification of blastocysts from infertile patients, egg donors and after IVM. Reprod Biomed Online. 2009;19(5):700‐707. 20021718 10.1016/j.rbmo.2009.09.011

[22] Dal Canto M , Moutier C , Brambillasca F , et al. The first report of pregnancies following blastocyst automated vitrification in Europe. J Gynecol Obstet Hum Reprod. 2019;48:537‐540. 31077870 10.1016/j.jogoh.2019.05.012

